# Cytokine Release Syndrome in COVID-19 Patients, A New Scenario for an Old Concern: The Fragile Balance between Infections and Autoimmunity

**DOI:** 10.3390/ijms21093330

**Published:** 2020-05-08

**Authors:** Andrea Picchianti Diamanti, Maria Manuela Rosado, Claudio Pioli, Giorgio Sesti, Bruno Laganà

**Affiliations:** 1 Department of Clinical and Molecular Medicine, Sant’Andrea University Hospital, Sapienza University of Rome, 00182 Rome, Italy; giorgio.sesti@uniroma1.it (G.S.); bruno.lagana@uniroma1.it (B.L.); 2 Research Consultant in Immunology, 00100 Rome, Italy; mariamanuelamrosado@gmail.com; 3 Laboratory of Biomedical Technologies, Division of Health Protection Technologies, Ente per le Nuove Tecnologie, L’energia e l’Ambiente (ENEA), 00196 Rome, Italy; claudio.pioli@enea.it

**Keywords:** SARS-CoV-2, COVID-19, rheumatoid arthritis, cytokine release syndrome, autoimmunity, immunomodulation, tocilizumab, hydroxychloroquine, baricitinib

## Abstract

On 7 January 2020, researchers isolated and sequenced in China from patients with severe pneumonitis a novel coronavirus, then called SARS-CoV-2, which rapidly spread worldwide, becoming a global health emergency. Typical manifestations consist of flu-like symptoms such as fever, cough, fatigue, and dyspnea. However, in about 20% of patients, the infection progresses to severe interstitial pneumonia and can induce an uncontrolled host-immune response, leading to a life-threatening condition called cytokine release syndrome (CRS). CRS represents an emergency scenario of a frequent challenge, which is the complex and interwoven link between infections and autoimmunity. Indeed, treatment of CRS involves the use of both antivirals to control the underlying infection and immunosuppressive agents to dampen the aberrant pro-inflammatory response of the host. Several trials, evaluating the safety and effectiveness of immunosuppressants commonly used in rheumatic diseases, are ongoing in patients with COVID-19 and CRS, some of which are achieving promising results. However, such a use should follow a multidisciplinary approach, be accompanied by close monitoring, be tailored to patient’s clinical and serological features, and be initiated at the right time to reach the best results. Autoimmune patients receiving immunosuppressants could be prone to SARS-CoV-2 infections; however, suspension of the ongoing therapy is contraindicated to avoid disease flares and a consequent increase in the infection risk.

## 1. Introduction

On 7 January 2020, after a cluster of cases with pneumonia of unknown origin in Wuhan, Hubei, China, researchers isolated a novel coronavirus, then called SARS-CoV-2 [1], and COVID-19, the induced disease.

SARS-CoV-2 is believed to have zoonotic origins (probably from the Huanan seafood market in Wuhan) and has close genetic similarity (96%) to a bat coronavirus, suggesting it emerged from a bat-borne virus [2]. An intermediate animal reservoir such as the pangolin is also thought to be involved in its introduction to humans, but efforts to identify intermediate hosts seem to have been unsuccessful [3,4]. Since the outbreak, thanks to its contagiousness/virulence characteristics and boosted by globalization, SARS-CoV-2 moved around the world faster than a virus has ever done before.

After three months since its appearance, it reached 1.5 × 10^6^ infected patients, with 3.5 × 10^5^ recovered patients and 100,000 deaths across the world [4]. Due to a number of unfortunate variables, Northern Italy was the first country outside of Asia affected by the rapid spreading of SARS-CoV-2, which led, at the end of March, to the highest number of deaths worldwide [4]. Typical clinical manifestations of the disease generally begin after less than 5–7 days of incubation and consist of fever, cough, fatigue, and mild dyspnea.

However, data from China reported that about 15–20% of patients have severe disease with interstitial pneumonia, which can progress to acute respiratory distress syndrome (ARDS) [5,6]. Pneumonia includes decreased oxygen saturation, with severe bilateral ground glass abnormalities, patchy consolidation, and alveolar exudates [5,6]. In patients with ARDS, the virus can induce an excessive and aberrant host immune response characterized by an upregulation of pro-inflammatory cytokines, resembling the clinical and serological features of cytokine release syndrome (CRS). CRS is a life-threatening emergency associated with high mortality; thus, an early identification is essential. 

At the moment, there are no specific antiviral treatments recommended for SARS-CoV-2 and no vaccine is currently available. A growing awareness of SARS-CoV-2 infection and CRS has led to exploring the use of immunomodulatory drugs as a potential treatment for the management of these patients.

Here, the complex relationship between infections and autoimmunity in the emergency scenario of the SARS-CoV-2 pandemic is discussed. We critically review the rationale for the adoption of immunosuppressive agents, commonly used in autoimmune diseases, in the treatment of SARS-CoV-2 infection and report current knowledge of ongoing studies.

## 2. SARS-CoV-2 Infection and the Pathophysiology of Cytokine Release Syndrome

CRS is a systemic inflammatory life-threatening condition typically associated with biological drug products, but also occurring during the response to some infections [7]. Initially described as a reaction to the use of the anti-CD3 (OKT3) monoclonal antibody (mAb) [8], this syndrome is the result of a cytokine storm. CRS has been under tight evaluation since it caused the admission to the intensive care unit of six healthy individuals enrolled in the phase I trial for the anti-CD28 mAb TGN1412A in 2006 [9]. CRS has been also observed in patients treated with immune checkpoint inhibitors or T cell therapy (CAR T cells) [10]. It is noteworthy that the cytokine storm due to massive T cell stimulation is also considered a relevant mechanism to the H5N1 influenza pathogenesis [11]. CRS and sepsis share several symptoms, and patients with CRS are at a high risk of infections, not only for the immunosuppressive treatments, but likely also for the CRS-associated immune dysregulation and tissue damages, especially at the mucosa barrier. Indeed, in CRS patients, infections principally involve the respiratory tract.

The exacerbated reaction to infections or to biological therapy is caused by the rapid recruitment of macrophages and neutrophils, which produce pro-inflammatory cytokines and alter the fragile balance between a controlled immune response and a host-damaging reaction. Damaged tissues release molecules normally not present outside the cells, including high-mobility group box 1 (HMGB1), ATP, uric acid, and DNA, further amplifying inflammatory responses. All these molecules are part of the so-called damage-associated molecular patterns (DAMPs). It is noteworthy that pathogen-associated molecular patterns (PAMPs) and DAMPs are recognized by the same group of innate immunity receptors, namely the pathogen recognition receptors (PRRs), which include toll-like receptors (TLRs), highly expressed in neutrophils and macrophages. Engagement of TLRs and other PRRs leads to further activation of NF-ĸB and the release of cytokines (IL-6, TNFα, IL-1, etc.) and other mediators of inflammation.

A relevant biological role has also been proposed for ferritin in CRS-related conditions, as well as in autoimmune disease has been proposed. The presence of hyperferritinemia, indeed, is a well-known feature in patients with different autoimmune conditions such as RA, systemic lupus erythematosus (SLE), and anti-phospholipid syndrome [12]. It has been supposed that several mechanisms involving the inhibition the H-ferritin-mediated suppression of immune cells may favor the loss of tolerance and the onset of autoimmunity [13]. Ferritin can be also a pro-inflammatory signaling molecule, and hyperferritinemia has been associated with different CRS-related conditions such as macrophage activation syndrome (MAS) and septic shock [14].

Ferritin could exert a pathogenic role in these diseases rather than being just the result of hyperinflammation. In fact, ferritin synthesis is mediated, not only by iron availability, but also by IL-1, IL-6, and TNF [15,16], which are overexpressed during CRS; on the other hand, it can induce the expression of pro-inflammatory cytokines, thus becoming part of a vicious loop [17]. 

In particular, it has been hypothesized that during an ongoing infection, the signaling mediated by bacterial/viral CpG DNA and TLR9 could activate the inflammasome, leading to IL-1 and IL-18 production [18,19,20]. Through DAMP signaling, infection could also increase the production of hemoglobin [21] and activate macrophages, which are prominent producers of ferritin [22,23], amplifying the inflammatory loop [23]. Moreover, a correlation between the serum levels of CD163, a marker of macrophage activation [24], and ferritin in patients with MAS has been reported [25]. Different therapeutic approaches could be useful in blocking this pathway at different levels, such as plasma exchange, intravenous immunoglobulins, or mAbs against IL-1 and IL-18 [18].

In patients affected by 2002/2003 SARS-CoV, immune dysregulation induced an abnormal inflammatory cytokine production by alveolar macrophages with a concomitant T cell dysfunction, involving both CD4 and CD8 T cells [26]. SARS patients with a more severe disease displayed higher serum levels of pro-inflammatory cytokines (IFN-γ, IL-1, IL-6) and chemokines (including IL-8) [27]. 

In CRS, IFN-γ further activates immune cells, especially macrophages, which are induced to produce more inflammatory cytokines and upregulate costimulatory ligands, feeding the harmful positive loop of inflammation. It is important to remember that type I interferons (IFNs) play a critical role in the normal/physiologic immune response to viruses by enhancing the toxic effects of CD8 T cells [28], activating NK cells, and restricting viral pathogenicity to the lung microenvironment. IFNαR-deficient mice infected with the H5N1 or the 1918 influenza virus show indeed higher mortality than wild-type mice, which display systemic dissemination of the virus [29]. It is noteworthy that some studies showed that coronaviruses, in particular MERS-CoV, can suppress the expression of both type I and type III IFNs, evading innate immune response and contributing to its pathogenicity [30]. In in vitro models, IFN-λ showed effects against 2002/2003 SARS-CoV and MERS-CoV, suggesting a possible use to control viral infections by coronavirus. Interestingly, the expression of the type III IFN receptors is more restricted to specific cell types (neutrophils and B cells) compared with type I IFN receptors, a feature that could restrain inflammatory response to the (initial) site of infection. In an animal model, treatment with IFN-λ2/IL-28A reversed the development of collagen-induced arthritis, also indicating an anti-inflammatory role in autoimmune responses [31]. However, in the contest of CRS associated with COVID-19, it cannot be excluded that type III IFN receptors are upregulated also in other cell types and that these cytokines could also contribute to the complex pathogenic process [32].

In SARS-CoV-2 patients with a worse prognosis, IL-6, IL-10, and TNF-α quickly rise and reach high levels. Conversely, in patients with milder symptoms, these cytokines reach lower levels, with their expression rising and declining during the illness and recovery phase, respectively [33]. 

The production of IL-10, an anti-inflammatory cytokine, in the context of CRS is often associated with the downregulation of neutrophil and monocyte function, a phenomenon termed immunoparalysis [34]. Although conceptually beneficial, the persistent downregulation of HLA-DR on monocytes, after sepsis, as well as after CRS, leads to higher mortality rates, suggesting that the recovery from immunoparalysis is critical for patient survival [35]. 

As above mentioned, systemic cytokines are considered to be massively produced by macrophages in SARS-CoV-2 patients [36]. However, also endothelial cells play a relevant role in CRS, not only as cells that are damaged by pathogens and inflammatory responses, but also as co-culprits. Endothelial cells indeed produce inflammatory cytokines, including IL-6, and upregulate adhesion molecules, further promoting leukocytes’ recruitment and capillary leakage [37]. 

In line with lymphocyte recruitment into the place of infection/inflammation, two recent studies showed that in patients with severe COVID-19, T cell lymphopenia is accompanied by an alteration in the distribution of circulating T cell subpopulations; patients, indeed, have increased frequencies of naive helper T cells and a reduction in memory helper T cells [38,39]. 

The cytokine unbalance associated with the response to SARS-CoV-2 could also affect the effectiveness of the immune response both in terms of viral clearance and future immune protection. For some of the recovered patients, protective antibodies have been described. They are mainly directed towards the receptor binding domain of the spike protein, and most likely, they interfere with viral entry [40]. Even if the majority of patients recover, some of them after discharge from the hospital, asymptomatic and negative RT-PCR viral RNA tests remain/return positive or even relapse [41]. Due to the limited number of described cases, incomplete virus clearance rather than lack of protection cannot be ruled out. Noteworthy, previous studies on 2002/2003 SARS-CoV showed heterogeneous results on B and T cell memory [42,43,44]. Information on the long-term quality of immune response, T and B cell immunological memory, and long- versus short-lived plasma cells towards SARS-CoV-2 is not available yet.

Altogether, these recent findings point to a major role of the host immune response, particularly of CRS, as a determining co-factor in the severe life-threating form of COVID-19. Why some patients develop an effective immune response, which is protective and not pathogenic, and why others have a non-protective life-threatening immune response is a key question. It is likely that genetic background, which is also involved in inflammatory responses, immune-mediated diseases (including autoimmunity), and co-morbidities, may not only weaken the host, but also may share “common” pathways in inflammatory damaging responses.

## 3. Anti-Host Therapy in SARS-CoV-2 Patients

Treatment of CRS involves the use of both antiviral agents to control the underlying infection and immunosuppressants to lower the aberrant pro-inflammatory response of the host.

For a better understanding, it is essential to remind about the natural course of SARS-CoV-2 infection.

**Figure 1:**
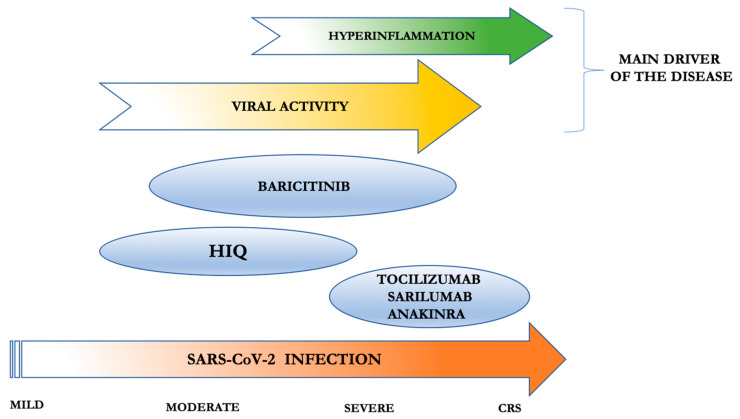
Use of anti-rheumatic immunosuppressive agents in COVID-19 patients, targeted to their immunomodulatory/antiviral activity and disease severity. Tocilizumab, sarilumab, and anakinra have the strongest immunosuppressive effect and have been already tested in cytokine release syndrome (CRS) (with tocilizumab being the only scheduled); thus, they should be administered in severe COVID-19 patients at the first manifestations of hyperinflammation. Baricitinib has both immunosuppressive effects (however, no data are available for CRS) and antiviral activity; thus, it could be adopted in the moderate/severe form of COVID-19. Hydroxychloroquine (HIQ) has antiviral properties and milder immunosuppressive activity than the other drugs; thus, it could be used in the moderate/severe form of COVID-19.

After the incubation period, indeed, the virus induces flu-like symptoms typical of mild disease; in some patients, the infection can progress to interstitial pneumonia (moderate disease) or severe pneumonia requiring oxygen therapy (severe disease), through to ARDS with respiratory failure [45]. This is probably the moment at which the shift from a controlled immune response to a host-damaging reaction begins to manifest clinically. Then, SARS-CoV-2 does not directly induce tissue damage, whereas the hyperinflammatory immune activation of the host becomes the effective protagonist of the disease. The early identification of this specific moment of transition is of key importance, to allow timely immunomodulatory intervention, thus achieving a tailored approach and the best therapeutic effects (Figure 1).

To that end, several serologic markers have been proposed, such as the presence of thrombocytopenia, lymphopenia, and increased levels of D-dimer and ferritin [46].

Some authors have also reported a role for IL-6 and IL-10 in monitoring COVID-19 patients. In particular, significantly higher levels of IL-6 and IL-10 have been identified in severe COVID-19 patients than in the milder forms, thus suggesting that these cytokines can be used to predict the transition from mild to severe infection [47].

## 4. Chloroquine and Hydroxychloroquine

Hydroxychloroquine (HIQ) is an orally administered and low-cost drug widely used as a monotherapy in clinical rheumatological practice mainly to treat the mild form of rheumatoid arthritis (RA), SLE, and Sjogren’s syndrome patients, as well as in combination therapy with conventional immunosuppressants, in more severe patients. HIQ has a pleiotropic activity ranging from immunomodulatory effects, to anti-thrombotic action and antiviral properties. The immunomodulatory activity of HIQ has been demonstrated in vitro to be exerted by several mechanisms.

HIQ interferes with lysosomal activity, impairing lysosomal and autophagosome functions and subsequently immune activation [48]. It can inhibit TLR-7 and TLR9 [49] signaling pathways and decrease the secretion of pro-inflammatory cytokines (IL-6, TNF-α, IL-1, IFN-γ) [50]. The anti-thrombotic mechanism is still poorly clarified; anyway, it has been reported that it can reverse platelet activation and reduce anti-phospholipid (aPL) antibody titers in aPL patients [51]; it can also improve endothelial dysfunction and reduce the expression of adhesion molecules such as VCAM-1 and E-selectin [52], mechanisms that could be relevant in severe COVID-19.

Regarding the antiviral effects, it is known that HIQ is able to block the infection of different viruses, including SARS-CoV-1, by increasing endosomal pH and by interfering with the glycosylation of the cellular receptor [53,54]. 

Several in vitro studies have been conducted to explore its efficacy in blocking SARS-CoV-2 infection. Chloroquine was found to inhibit the virus at a low micromolar concentration, with a half maximal effective concentration (EC_50_) of 1.13 μM and a half cytotoxic concentration (CC_50_) greater than 100 μM, which can be achievable with the standard dosing regimen [55]. In a recent study, HIQ was found to have a higher antiviral effect than chloroquine, with a lower EC_50_ [56]. 

As a consequence of these encouraging results, at least 23 clinical trials are being carried out to evaluate the efficacy and safety of chloroquine or HIQ in the treatment of SARS-CoV-2 patients [57]. By now, the results have demonstrated efficacy in reducing the exacerbation of pneumonia, improving lung imaging findings, and promoting a virus-negative conversion without serious adverse events [58]. A guideline document promoted by the Italian Society of Infectious and Tropical Disease recommends the use of chloroquine 300 mg × 2/day or HIQ 200 mg x 2/day, in patients presenting with mild respiratory symptoms and comorbidities, as well as in patients with severe respiratory failure [59].

## 5. IL-6 Inhibitors

IL-6 is a pleotropic cytokine with several immunological activities. It plays a role in the differentiation of mature B cells into plasma cells, and combined with TGF-β, it induces the differentiation of naive CD4 positive T cells into Th17-cells and induces the production of acute-phase proteins such as CRP, fibrinogen, serum amyloid A, and hepcidin [60,61]. In bone marrow, IL-6 induces the maturation of megakaryocytes into platelets and the activation of hematopoietic stem cells [60,61].

Tocilizumab and sarilumab are a humanized and human mAb, respectively, recognizing the soluble and membrane-bound forms of the IL-6 receptor. They are part of the first line biological therapy in patients with moderately to severely active RA and juvenile idiopathic arthritis; more recently, tocilizumab has been scheduled also for giant cell arteritis patients [61]. 

It is of note that IL-6 in combination with TGF- β is also produced by fibroblasts and activated macrophages exerting a pro-fibrotic effect at different sites such as lungs, skin, and liver [61]. 

In a recent work, we proposed tocilizumab as a valid therapeutic strategy in patients with interstitial pneumonitis associated with RA, because it might contrast the pro-fibrotic effects of IL-6, ameliorating both the articular and lung involvement [62]; this observation was recently confirmed by other authors [63]. As reported above, in some patients, SARS-CoV-2 infection can induce an uncontrolled and aberrant host hyperimmune response that is associated with lung damage and fibrosis, leading to life-threatening multi-organ failure. Serologically, an increase in the serum concentrations of IL-1, IL-6, IL-2, IL-7, IL-10, TNF-α, granulocyte colony-stimulating factor, interferon-γ-inducible protein 10, monocyte chemoattractant protein 1, and macrophage inflammatory protein 1-α has been found [64,65,66]. Furthermore, different Chinese authors reported that a lower lymphocyte count and elevated CRP, ferritin, D-dimer, and IL-6 were poor prognostic factors in SARS-CoV-2 patients [67].

The efficacy of tocilizumab in resolving life-threatening CRS during CAR T cell therapies was assessed in small patient cohorts [7]; however, the striking positive results led to its rapid approval for the treatment of CRS by the FDA in 2017 followed by EMA in 2018 [68,69]. Based on these data, small retrospective studies on patients affected by severe COVID-19 demonstrated that tocilizumab improved CT scan ground glass lesions and oxygen saturation, normalized CRP levels, and lymphocyte count in a significant percentage of patients [70,71].

IL-6 inhibitors should be initiated at the early stages of hyperinflammation, after discussion between critical care medicine and hematology/rheumatology and infection specialists; one additional dose may be considered if clinical deterioration persists (max two doses per course in severe SARS-CoV-2) [72,73]. 

A multicenter randomized clinical trial (RCT) of tocilizumab has been approved in China and is currently ongoing in patients with SARS-CoV-2 pneumonia and elevated IL-6 levels (ChiCTR2000029765).

Several clinical trials on the use of tocilizumab in patients with COVID-19 are already posted on ClinicalTrials.gov. These trials are enrolling different sets of the COVID-19 population ranging from patients with recent onset pneumonia to life-threatening-associated CRS.

There is also heterogenicity among the primary outcomes, but those mainly used are the proportion of subjects with normalization of fever and oxygen saturation at 14 days, the proportion of patients requiring mechanical ventilation and intensive care unit (ICU) admission, and the one-month mortality rate. The increase in lymphocyte count, decrease in CRP, and amelioration of CT lung opacity are frequently reported secondary endpoints (NCT04317092).

Another five trials have been posted on ClinicalTrials.gov on the use of sarilumab in COVID-19 patients, three of which have started recruitment. Two studies are enrolling hospitalized COVID-19 patients aiming at evaluating the safety and effectiveness of low or high dose i.v. sarilumab (NCT04315298; NCT04327388). The other study is recruiting patients with moderate/severe pneumonia associated with SARS-CoV-2, and the primary endpoint is the survival without the need for ventilator utilization at Day 14 (NCT04324073).

## 6. IL1-Inhibitors

In addition to IL-6, also IL-1 plays an important role in CRS [74,75]. IL-1β and IL-1α increase acute-phase signaling, homing of immune cells to the site of primary infection and epithelial cell activation, both inducing the production of many other cytokines [75,76]. IL-1β can also drive proinflammatory activity in the respiratory tract as shown by its presence in the bronchoalveolar lavage fluid of patients with lung injury [76].

Anakinra is a 17 kD recombinant, non-glycosylated human IL-1Ra that blocks IL-1α and IL-1β. It was approved for treating RA, cryopyrin-associated periodic syndromes, and Still’s disease [77]. 

It has been reported to be safe and effective in the management of sepsis-associated MAS, in particular those with increased liver enzymes, hypofibrinogenemia, and thrombocytopenia [78]. Conversely, the other anti-IL1β inhibitor, canakinumab, has not been demonstrated to be beneficial in MAS [79]. As of now, four clinical trials are recruiting patients with COVID-19, severe acute respiratory failure, and CRS, aiming at evaluating the safety and effectiveness of anakinra alone or in combination with anti-IL-6 agents (NCT04330638, NCT0432402, NCT04357366, NCT04339712).

## 7. JAK-STAT Inhibitors

Janus kinase inhibitors, also known as JAK inhibitors, are a class of orally administered targeted synthetic immunosuppressants that act by inhibiting the activity of one or more of the JAK family members (JAK1, JAK2, JAK3, TYK2), thereby interfering with the JAK-STAT signaling pathway. Several inflammatory cytokines, involved in autoimmunity diseases, by binding to their receptors, initiate a JAK dependent phosphorylation cascade constituting the signaling pathway of gene transcription. Hence, drugs that inhibit the activity of JAK block cytokine signaling.

These inhibitors have therapeutic application in the treatment of cancer, RA, psoriasis, psoriatic arthritis, and inflammatory bowel diseases [80]. 

By now three JAK inhibitors are approved for the treatment of rheumatic conditions, tofacitinib, baricitinib, and upadacitinib. Tofacitinib is a specific inhibitor of JAK3 and to a lesser extent JAK1 and JAK2. Baricitinib reversibly inhibits JAK1 and JAK2, with moderate activity against TYK2 and significantly less against JAK3, whereas upadacitinib is a selective inhibitor of JAK1 [81]. 

Pharmacology studies, in vitro, demonstrated that all three anti-JAK antibodies can inhibit JAK1/2-dependent cytokines (IL-6 and IFN-γ) and the JAK1/TYK2-dependent cytokines (IL-10 and IFN-α), whereas tofacitinib and upadacitinib are the most potent inhibitors of the JAK1/3-dependent cytokines (IL-2, IL-4, IL-15, and IL-21) [81]. Considering the above data, the adoption of these drugs for CRS management could be useful. Indeed, preclinical studies on murine models of Hemophagocytic lymphohistiocytosis (HLH) and MAS showed the efficacy of JAK inhibition [82,83].

JAK inhibitors have also gained the attention of researchers in the scenario of SARS-CoV-2 infection, for their demonstrated antiviral properties. Most viruses enter cells through receptor-mediated endocytosis. One of the known regulators of endocytosis is the AP2-associated protein kinase 1 (AAK1); thus, AAK1 inhibitors can interrupt the passage of the virus into cells and can be helpful in preventing virus infections [84]. Among AAK1 inhibitors, baricitinib has shown the highest affinity, being able to inhibit AAK1 at the standard therapeutic dosage for RA [85]. Furthermore, the use of baricitinib appears to be particularly safe also in combination with antiviral drugs considering its minimal interaction with the CYP drug-metabolizing enzymes [86]. Tofacitinib showed no detectable inhibition of AAK1, whereas no data are yet available on the effect of upadacitinib on AAK1 [87].

Currently an Italian trial is recruiting patients with mild to moderate SARS-CoV-2 infection (NCT04320277). Patients will be treated with a combination of baricitinib and antiviral therapy with ritonavir. The primary endpoint of the trial is the percentage of patients requiring transfer to ICU as compared with the rate of transfers observed in controls. Another European trial is starting recruitment of patients with moderate to severe SARS-CoV-2 infection; baricitinib will be used in monotherapy compared to lopinavir/ritonavir, HIQ, and IL-6 inhibitor (NCT04321993), all administered alone.

## 8. TNF-α Inhibitors

TNF-α belongs to a large family of cytokines known as the TNF superfamily. It is produced mainly by activated macrophages, NK, T, and B cells, and exerts its action through two receptors called TNFR1 and TNFR2. After binding to its receptors, TNF-α leads to a myriad and often conflicting effects reflecting complex cross-talk mechanisms [88]. It can mediate both apoptosis and cell activation, proliferation of B cells, and enhancement of cytotoxic activity of NK cells. The NF-κB activation pathway following TNF-α binding also induces the production of pro-inflammatory cytokines such as IL-4, IL-6, and IL-8, leading to extensive tissue damage such as vascular leakage and lung injury seen in many chronic inflammatory diseases [89,90]. As for IL-6, TNF-α is responsible for systemic inflammatory manifestations such as fever and cachexia and has been shown to be a central cytokine in the activation and maintenance of CRS [75]. TNF-α is a potent antiviral cytokine that acts directly by killing the virus-infected cells prior to maximal virus replication. However, it is known that the viral spike protein of SARS-CoV-2 is able to induce a TNF-α-converting enzyme (TACE)-dependent shedding of the ACE2 ectodomain, which is coupled to TNF-α production and is crucial for the penetration of the virus into the cell [90,91]. Based on these observations, TNF-α appeared to be an attractive therapeutic target. Treatment of MAS patients with etanercept (a fusion protein made from the combination of two soluble human 75k TNF-R linked to an Fc portion of an IgG1) has already been described, but data are scarce and contradictory [92,93]. A trial evaluating the efficacy and safety of adalimumab (human mAb directed against TNF-α) in SARS-CoV-2 patients with severe respiratory failure and CRS has recently been registered in the Chinese Clinical Trial Registry (ChiCTR2000030089).

## 9. Other Potential Immunological Therapeutic Options

### 9.1. B Cell Inhibitors

Rituximab (RTX) is a chimeric anti-CD20 mAb, approved for the treatment of B cell malignancies, RA, and ANCA-associated vasculitis [94,95,96].

B cell ablation with rituximab has been observed to have efficacy in macrophage activation syndrome in patients with underlying Epstein-Barr virus (EBV) [97]. The proposed mechanism is the reduction of viral load via destruction of the reservoir of EBV-infected cells. Unfortunately, RTX therapy has been shown to induce CRS, probably caused by the rapid destruction of tumor cells and consequent changes of serum cytokine levels [98]. It seems that CRS is a side effect of RTX therapy considering that it occurs mainly in patients with a very high tumor burden. Thus, its use appears not to be indicated in CRS secondary to SARS-CoV-2 infection. 

### 9.2. T Cell Modulation Therapy

CRS occurring during SARS-CoV-2 infection has a clinical and serological profile resembling that of secondary HLH. Given the central role of CD8 T cells in secondary HLH, non-ablative inhibitors of T cell function are also attractive therapeutic choices [74]. 

Cyclosporine (CyS) is a cyclic undecapeptide that binds intracellularly to cyclophilin and suppresses calcium-dependent phosphatase calcineurin pathway activation. Functional consequences are the block of T cell survival and activation and inhibition of IL-2 production [99,100]. 

It is used for the prevention of transplant rejection, as well as in different autoimmune conditions such as RA, psoriasis, and glomerulonephritis [101]. It has already been adopted as part of the standard protocol in familiar HLH patients [102]. 

It could also be relevant to underline that CyS is able to inhibit in vitro the function of a transmembrane protein called P-glycoprotein that pumps out of the cell several drugs, including amprenavir, indinavir, nelfinavir, ritonavir, and saquinavir, thus being crucial in the development of drug resistance to anti-retroviral therapy. CyS has also been demonstrated to revert in vivo drug resistance to methotrexate in RA and Psoriatic Arthritis (PsA) patients [103,104].

On the basis of the anti-inflammatory activity and its ability to improve the effectiveness of antiviral therapy, low dose CyS in combination with antiviral drugs might be rational in selected patients without severe renal involvement. 

Abatacept is a dimeric fusion protein composed of the human CTLA-4 extracellular domain and a human FcIgG1 that binds with high-affinity CD80/CD86 molecules, thus impairing T cell activation and T-B cell cross-talk during the immune response [103,105]. It is scheduled for use in RA and more recently PsA patients. Abatacept has been proposed as a valid option for interstitial lung disease associated with RA in several case series [106] and has already been proven in a few cases of MAS refractory to standard intervention, demonstrating mild effectiveness and safety [107]. 

The main data on the use of anti-rheumatic drugs currently tested in clinical trials of COVID-19 patients are resumed in Table 1.

## 10. Autoimmunity and SARS-CoV-2 Infection

As previously stated, SARS-CoV-2 infection represents an emergency scenario of an old challenge, which is the complex and interwoven link between infections and autoimmunity. This complex link has implications at the biological level in terms of individual susceptibility/resistance, as well as in the delicate balance to be reached with therapeutic options. Polymorphisms in the HLA locus have been shown to affect individual susceptibility with variants that confer resistance to some viral infections and predispose to autoimmune diseases and others that show more complex associations increasing the risks for both autoimmunity and infections [108]. Susceptibility to several infectious diseases including HIV, hepatitis B, and influenza is associated with specific HLA haplotypes. For instance, HLA-A*11, HLA-B*35, and HLA-DRB1*10 have been shown to correlate with susceptibility to influenza A (H1N1) infection. It would be important therefore to understand if specific HLA loci are associated with susceptibility to SARS-CoV-2 or to the development of a protective immune response. While it is still early to have information on SARS-CoV-2 and HLA, studies on 2002/2003 SARS-CoV did not show associations with HLA-A, HLA-B, and HLA-DRB1 allele frequencies [109], whereas some variants of HLA-DRB1 seem to correlate with susceptibility to MERS [110]. Noteworthy, some HLA-DRB1 amino acid variants are associated with RA, conferring either susceptibility or resistance to this disease [108].

Patients with autoimmune diseases are, indeed, at high risk of infections, due to endogenous (dysfunctional immune system) and external factors (i.e., immunosuppressants). 

In RA patients, the risk for infections is about double with respect to healthy individuals, and they are mainly located at the bone and joints, skin, soft tissues, and respiratory levels [111]. In RA, patient data on infection risk generally show that methotrexate (the gold standard immunosuppressants for inflammatory arthritis) and HIQ are the therapies impacting the least in the increased susceptibility to infection, both being considered relatively safe [112,113]. The risk of infections observed in RA patients treated with biologic drugs is generally reported to be higher compared with patients receiving conventional immunosuppressants [114,115]. In a retrospective observational cohort-study, our group evaluated the role of methotrexate, corticosteroids, and TNF-α antagonists alone or in combined therapy on non-serious and serious infections in RA and spondyloarthritis (SpA) patients. We identified an incidence ratio/100 patient-years of 36.3 for all infections, being 34.9 for non-serious and 1.4 for serious infections [116]. These results are similar to those reported from the CORRONA Register on a larger RA U.S. patient population [117]. As confirmed by other authors, we also found that the combination of anti-TNF-α with corticosteroids was the most pro-infective treatment, whereas methotrexate alone was relatively safe [116,117,118,119].

The corticosteroids/anti-TNF-α combination can indeed synergize in lowering TNF-α levels through different and independent mechanisms, with the consequent increase of the anti-inflammatory effect, but at the expense of a rise in the risk of infection [118,119]. As reported by metanalyses and real-life studies, among biological agents, abatacept seems to be the safest in terms of infectious risk [120,121]. Autoimmune diseases and infections are already linked with alteration in disease activity. 

In autoimmune patients, indeed, infections may induce disease flare-up that may be followed by a severe clinical course, representing a frequent cause of death (20–55%) [122]. 

On the other hand, it is known that a higher disease activity is associated with a higher probability of developing infections [117]. In fact, high disease activity is the result of chronic inflammation against self, which can exhaust the immune resources and deviate the immune response from the danger signals delivered by pathogens [123]. Conversely, infections may stimulate the immune system, thus leading to a reactivation of the underlying autoimmune disease. 

In view of the above, it is not surprising that, although autoimmune patients under immunosuppressive agents could be prone to SARS-CoV-2 infections, suspension of the ongoing conventional and biological therapy is contraindicated to avoid disease flares with a consequent increased risk of infection [124].

It could be hypothesized that the impairment of immune response caused by the ongoing therapy could be a double-edged sword. On the one hand, indeed, immunosuppression increases the risk and consequently the prevalence of SARS-CoV-2 infections in autoimmune patients, but on the other hand, it could decrease the risk of the aberrant hyperinflammatory response seen in patients with SARS-CoV-2. A registry of COVID-19 in autoimmune patients has been created by the Italian Society of Rheumatology. This registry could give useful information to clarify the above hypothesis and be of crucial help for therapeutic decision-making in this particular group of patients. Whatever these results will show, one should keep in mind that co-infection can often be lethal, and thus, the use of anti-influenza and anti-pneumococcal vaccinations, already recommended in these patients [125], assumes even more importance in this new scenario of the COVID-19 pandemic.

## 11. Conclusions

Several trials evaluating the safety and effectiveness of immunosuppressants commonly used in autoimmune patients are ongoing in patients with COVID-19 and CRS, some of which are achieving promising results. However, such a use should follow a multidisciplinary specialist approach, be accompanied by close monitoring, be tailored to patient’s clinical and serological features, and be initiated at the right time to reach the best results. It is also important to take into account that these drugs, pivotal in the treatment of many autoimmune patients, are already running out; in fact, shortages are being reported from several European countries (i.e., i.v. tocilizumab and HIQ). Autoimmune patients under immunosuppressive drugs could be prone to SARS-CoV-2 infection; however, suspension of the ongoing conventional and biological therapy is contraindicated to avoid disease flares and the consequent increase in the infection risk.

## Figures and Tables

**Table 1:** Anti-rheumatic immunological drugs currently tested in clinical trials of COVID-19 patients.

	Hydroxychloroquine/Chloroquine	Tocilizumab/Sarilumab	Anakinra	Baricitinib	Adalimumab
**Mechanism** **of Action**	Immunomodulation = Impairing lysosomal functions Antiviral = Increasing endosomal pH and interfering with cellular receptors	Immunomodulation = IL-6 inhibition	Immunomodulation = IL-1 inhibition	Immunomodulation = IL-6 and IFN-γ inhibition Antiviral = AAK1 inhibition	Immunomodulation = TNF-α inhibition
**Target Population**	Mild with comorbidity Moderate/severe	Moderate/severe with or without CRS	Severe and CRS	Mild to severe, with or without CRS	Severe and CRS
**Main Therapeutic Regimen**	HIQ = 200–400 mg/day/orally CQ = 300 mg × 2/day/ orally	Single infusion 8 mg/kg/i.v. (max 800 mg) Second cycle after 8–12 h if no significant response **	100 mg/day/sc or 100 mg/4 times a day/i.v.	2–4 mg/day/orally	Not reported
**Main Safety Exclusion Criteria**	Consider drug-to-drug interactions (i.e., QT interval prolongation) Retinopathy Severe renal dysfunction	Active TB and infections other than COVID-19 Bowel diverticulitis Severe heart failure Neut < 500/mmc Plt < 50,000/mmc Pregnancy	Active TB and infections other than COVID-19 NYHA class III/IV Severe renal dysfunction Pregnancy	Active TB and infections other than COVID-19 History of thrombophlebitis Severe renal dysfunction Pregnancy	Active TB and infections other than COVID-19 NYHA class III/IV Severe renal dysfunction Pregnancy
**Specific Parameters to Closely Monitor**		Blood count (reduction in Neut and Plt), AST, ALT, procalcitonin *, IL-6	Blood count, AST, ALT, procalcitonin *	Blood count, AST, ALT, procalcitonin *	Blood count, AST, ALT, procalcitonin *

* To exclude active infections from sources other than COVID-19; ** sarilumab is being studied also at 11 mg/kg/i.v.; both tocilizumab and sarilumab are being tested also s.c. CRS = cytokine release syndrome, Plt = platelets, Neut = neutrophils, ALT = alanine transferase, AST= aspartate transferase, i.v. = intravenous, s.c. = subcutaneous, HIQ = hydroxychloroquine, CQ= chloroquine.
